# Acceleration of Intrusion Detection in Encrypted Network Traffic Using Heterogeneous Hardware †

**DOI:** 10.3390/s21041140

**Published:** 2021-02-06

**Authors:** Eva Papadogiannaki, Sotiris Ioannidis

**Affiliations:** 1 Institute of Computer Science, Foundation for Research and Technology—Hellas (FORTH), GR-70013 Heraklion, Crete, Greece; sotiris@ece.tuc.gr; 2 School of Electrical and Computer Engineering, Technical University of Crete, University Campus, GR-73100 Chania, Crete, Greece

**Keywords:** network intrusion detection, encrypted network traffic, encrypted network traffic inspection, network packet metadata, GPGPUs, integrated GPU, OpenCL

## Abstract

More than 75% of Internet traffic is now encrypted, and this percentage is constantly increasing. The majority of communications are secured using common encryption protocols such as SSL/TLS and IPsec to ensure security and protect the privacy of Internet users. However, encryption can be exploited to hide malicious activities, camouflaged into normal network traffic. Traditionally, network traffic inspection is based on techniques like deep packet inspection (DPI). Common applications for DPI include but are not limited to firewalls, intrusion detection and prevention systems, L7 filtering, and packet forwarding. With the widespread adoption of network encryption though, DPI tools that rely on packet payload content are becoming less effective, demanding the development of more sophisticated techniques in order to adapt to current network encryption trends. In this work, we present HeaderHunter, a fast signature-based intrusion detection system even for encrypted network traffic. We generate signatures using only network packet metadata extracted from packet headers. In addition, we examine the processing acceleration of the intrusion detection engine using different heterogeneous hardware architectures.

## 1. Introduction

The adoption of common encryption protocols—such as SSL/TSL and IPsec—has been recently growing, aiming towards the protection of network communication channels. Statistics report that, currently, more than 75% of the Internet traffic is encrypted, while this percentage is constantly rising [1,2]. Although this practice is crucial for the protection of end-users and services, it introduces new challenges for Internet traffic analysis and monitoring tools, which mostly rely on techniques like deep packet inspection (DPI). DPI is based on pattern matching, which enables searching for specific content inside the network traffic. Common applications for DPI include firewalls, intrusion detection and prevention systems, L7 filtering, and packet forwarding [3,4]. However, with the widespread adoption of network encryption protocols, solutions that rely on scanning packet payload contents are becoming less effective and new approaches have to be implemented in order to ensure their correct operation. Existing DPI solutions extract mostly coarse-grained information when operating on encrypted network traffic. Thus, it is important to adapt to current encryption trends to analyze fully encrypted network connections.

An approach to inspect encrypted network traffic is the generation of signatures based on packet metadata, such as the packet timestamp, size, and direction. These metadata can be usable even in encrypted traffic, since they can be easily extracted from packet headers. Recent related work has proven that revealing the traffic nature in encrypted communication channels is feasible using machine learning techniques either for network analytics, network security, privacy, or fingerprinting. More specifically, such works focus on investigating which network related characteristics reveal the nature of the network traffic. As it occurs, we can divide the resulted network-related characteristics that make traffic classification feasible—even in encrypted networks—into three groups: (i) time-related, (ii) packet-related, and (iii) statistical characteristics [5,6,7]. Based on the outcomes of those works, we build signatures from the most descriptive and revealing packet metadata categories; the sequence of packet payload lengths (ordered by the time the arrive at the network interface) and the packet direction. In this work, we focus on signature-based intrusion detection, and we process only network packet headers.

In addition, to cope with the ever increasing network speeds, we investigate the utilization of commodity hardware accelerators, such as GPUs, for high performance intrusion detection against network traffic. The benefits for such an implementation is the high processing throughput, as well as the low cost of powerful commodity high-end off-the-shelf GPUs (in contrast to expensive server setups). Since GPUs offer stream processing, real-time traffic inspection can be achieved [8,9,10,11,12]. Fast metadata matching can enhance the implementation of numerous applications tailored for encrypted networks, such as traffic monitoring and firewalls. Such system can be utilized by service providers for analytics and measurements in order to ensure quality of service for their clients. Our signatures that are extracted through an offline analysis phase are compiled into an Aho–Corasick automaton that enables simultaneous multi-pattern matching. The incoming network traffic is grouped into batches and then transferred to the device memory space. Our engine is able to report suspicious behavior during the pattern matching period against the incoming network traffic.

**Figure 1:**
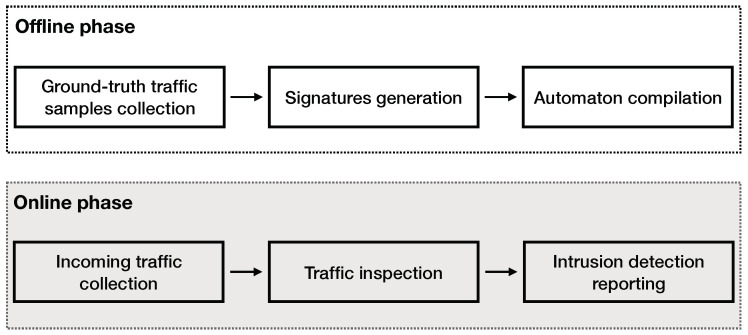
A high-level design overview of this work. In the offline phase, we (i) process the ground-truth dataset retrieved from [13], (ii) we generate signatures, and (iii) we build the automaton. In the online phase, we process the input traffic using our intrusion detection engine that reports any suspicious activity identified by our signatures.

A high-level overview of this work is presented in Figure 1. This work can be divided into two parts—the offline and the online phase. In the offline phase, we firstly collect and process the ground truth samples. The ground-truth samples used for this work are collected from the publicly available dataset UNSW-NB 15 [13]. The generated signatures are compiled into an Aho–Corasick automaton. In Section 3, we discuss the details of the automaton implementation. In the online phase, our system processes the input traffic and inspects the flows contained, reporting suspicious activity, based on the signatures. Details on the implementation of our signature-based intrusion detection engine are discussed in Section 3.

The contributions of this work are the following: (This is an extended version of the paper “Head (er) Hunter: Fast Intrusion Detection using Packet Metadata Signatures” [14] that was published in the proceedings of the 2020 IEEE 25th International Workshop on Computer Aided Modeling and Design of Communication Links and Networks (CAMAD)):

- We generate signatures for intrusion detection using strictly packet metadata extracted from packet headers, making our engine suitable for encrypted network traffic. More specifically, we focus on packet payload size and packet direction.
- Aiming to advance the state-of-the-art, we propose and present the implementation of a signature-based intrusion detection engine tailored for packet metadata and not packet contents. Current state-of-the-art techniques examine only the feasibility of classifying traffic using packet metadata, without offering a real traffic inspection engine implementation.
- We evaluate our work in two parts: the (i) signature quality and (ii) pattern matching engine performance. Current state-of-the-art techniques examine only the accuracy of the proposed analysis techniques.
- We extend the most popular string searching algorithm, Aho–Corasick, to also support integers, for packet metadata matching. To improve the processing throughput of the intrusion detection engine, we use off-the-shelf GPUs and a commodity CPU.

## 2. Encrypted Traffic Signatures

In this section, we present the signature generation procedure. More specifically, we discuss our signature generation methodology, and then we detail the procedure of the ground-truth dataset manipulation that we followed to produce the signatures.

### 2.1. Encrypted Traffic Signature Generation

**Figure 2:**
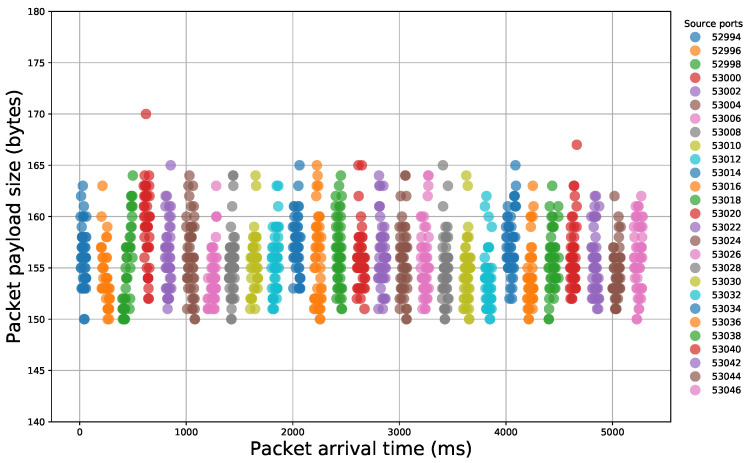
Illustration of packet payload size sequences within a network traffic capture of a file scanning attempt.

**Figure 3:**
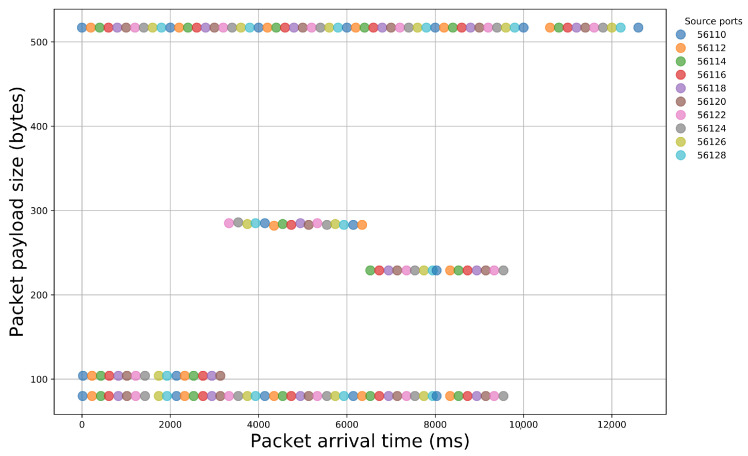
Illustration of packet payload size sequences within a network traffic capture of a login attempt to a web server.

After the examination of the literature and during our analysis, we observed that specific sequences of packet payload sizes reliably signify certain malicious activity. Indeed, if we illustrate packet traces that contain different events, we can easily notice the diversity that appears in packet size sequences (Figure 2 and Figure 3). Thus, network packet size is a very important feature that, if properly handled, can offer important insights for the nature of the underlying network traffic.

In this work, we aim for an expressive yet simple enough signature format to facilitate the automated generation of signatures. Another consideration for a practical system is to minimize the amount of state information that a deep packet inspection engine needs to maintain per flow in order to evaluate patterns across packet sequences in the same flow. The advantage of our solution is that it can be implemented with an automaton, without the need to retain previously observed packet sizes so as to support backtracking, and that it is expressive enough to capture the traffic metadata of interest. As already mentioned, we do not parse and we do not process any network packet payloads—we only focus on packet headers. This means that the only information that we have available is contained inside a packet header. In addition, we aim to avoid adding any complexity in the signature specification procedure and, thus, we decide to utilize only the following fields for each network packet: (i) source IP address, (ii) source port, (iii) destination IP address, (iv) destination port, (v) protocol, (vi) payload size, and (vii) packet timestamp. Afterwards, for each one of these network flows, we produce a sequence of metadata; (i) packet payload sizes and (ii) packet directions. Some examples of packet payload-size sequences that are used as signatures can be found in Table 1.

The second signature in Table 1 contains negative values. The sign of each number shows the direction of the packet when we produce signatures for bi-directional network connections. More specifically, a packet payload size with a negative sign indicates that the packet is received and not transmitted. This is a technique to add extra expressiveness on the signatures. To build the signatures, we use a fraction of the network packet traces dataset. More specifically, we build a single signature per record. This signature represents the actual sequence of packet payload sizes and has one of the two formats that we presented in the previous paragraph (i.e., with or without the sign).

To allow a more flexible format, we introduce a regular-expression-like format to present the signatures (Table 2). Our proposal can be extended for additional expressiveness, for example by adding other regular expression constructs such as groups or disjunctions. In this work, we kept the signature format complexity to a minimum to avoid complicating the signature generation process as well as the automaton creation. Disjunction, in particular, is handled by providing a disjunctive set of rules, instead of extending the language syntax with an embedded disjunctive operator. The downside is that a larger set of patterns may be required; however, we did not find this to be a problem in practice. Having retransmitted packets is an unpredictable network behavior, so we might lose a reporting solely due to a not properly defined upper bound in the repeat range of an expression. Thus, we choose to handle retransmitted TCP packets by discarding them in a packet filtering phase.

#### Fine-Grained Signatures

The network traffic ground-truth samples that we use to build our signatures provide fine-grained labels, which, apart from classifying a network flow into the two categories “*benign*” and “*attack*”, also distinguish the attacks into categories (e.g., DoS, reconnaissance). Thus, we use this fine-grained information offered through the ground-truth dataset in order to build signatures that will further point to the most probable attack type, besides solely classifying the network flow as malicious or not. In Section 4, we discuss the quality of the generated signatures with respect to their attack type.

### 2.2. Ground-Truth Dataset Manipulation

The first step to generate effective signatures is the analysis of a ground-truth dataset. For the purpose of this work, we utilize publicly available datasets (i.e., [15]) that contain network traces originated from malicious activities and, most importantly, these datasets are labeled and classified according to the attack type. As we discuss in the following paragraphs, this enables us to produce signatures able to report suspicious network flows in a fine-grained manner.

More specifically, in the dataset that we use, each record that represents a single attack contains a number of attributes. Some of these attributes are presented in Table 3. However, these attributes include only coarse-grained information that describe every network flow in the original dataset. In this work, we aim to build signatures based on packet-level information—something that is partially provided by these attributes. Thus, we use only a subset of these attributes that will eventually enable us to locate the actual network traffic flows inside the traffic traces that are also publicly available (in a .pcap format). As already mentioned, using a fraction of the attributes, we map every network flow to an attack type. More specifically, each network flow is characterized by the typical 5-tuple “*source IP address, source port, destination IP address, destination port, protocol*” combined by the two timestamps that indicate (i) the start and (ii) the termination of each connection. Then, we group these network flows by the attack type. Finally, a single record in our processed dataset is formatted as such: “Attack Record 1: 1421927416, 1421927419, 175.45.176.2, 149.171.126.16, 23357, 80, TCP, Exploits”, where 1421927416 is the first packet timestamp captured, 1421927419 is the last packet timestamp captured, 175.45.176.2 is the source IP address, 149.171.126.16 is the destination IP address, 23357 is the source port number, 80 is the destination port number, TCP is the connection protocol and, finally, Exploits is the attack category. Then, for each one of the records, we extract the whole network packet trace from the traffic, similar to a network flow.

**Figure 4:**
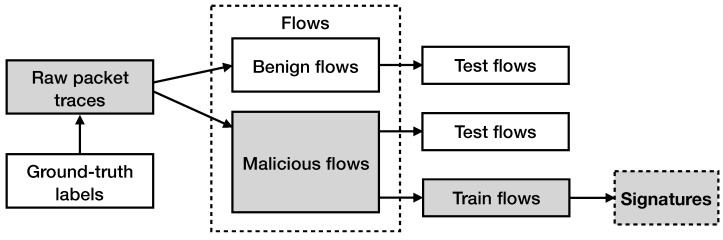
Overview of the signature generation procedure. The traffic traces are arranged into network flows. Each flow is marked as malicious or benign, according to the ground-truth labels. Malicious flows are further divided into two sets; the first set is used for testing and the second set is used for signature generation.

Figure 4 displays a high-level overview of the signature generation procedure that we follow. As previously mentioned, we break the raw network traffic traces into flows. Each flow is characterized by the corresponding label that occurs from the ground-truth records (i.e., benign or malicious flow). After having divided the resulted network flows into the two categories, we divide the malicious network flows into two sets, the one that contains the malicious network flows that will be used for testing (60% randomly selected flows) and the one that contains the malicious network flows that will be used to produce our signatures (40% randomly selected flows).

## 3. Intrusion Detection Engine

The second part of our solution is the implementation of the intrusion detection engine. Our intrusion detection system is signature-based. This means that every report of suspicious network flow is produced only when one, or more, of the predefined signatures is found inside the network traffic. The main process of signature-based systems is pattern matching. Inspired by one of the most popular algorithms for fast string pattern matching, called Aho–Corasick [16], our extended implementation operates on packet sizes, which are represented as short integers, instead of a typical Aho–Corasick implementation that operates on packet payloads, which are represented as strings. In the rest of this section, we describe the implementation details of our intrusion detection system.

### 3.1. Automaton

The Aho–Corasick algorithm is an efficient string searching algorithm that matches the elements of a finite set of strings against an input. It is able to match all pattern strings simultaneously, so its complexity does not depend on the size of the pattern set. It works by constructing an automaton that executes transitions for each 8-bit ASCII character of the input text. Since we aim to adapt the algorithm to match signatures that describe sequences of packet payload sizes, we replace the 8-bit characters with 16-bit values that represent packet sizes, similar to [6]. The algorithm builds a finite state machine, resembling a trie with added “failure” links between the trie nodes. These failure links are followed when there is no remaining matching transition, and they offer fast transitions to other branches of the trie that share a common prefix, without the need for expensive backtracking using earlier inputs. This way, the algorithm allows the interleaving of a large number of concurrent searches, such as in the case of network connections because the state of the matcher can be preserved across input data that are observed at different points in a time period by storing a pointer to the current state of the automaton, with the state maintained for each connection. Otherwise, backtracking would require the maintenance of expensive per-flow state for previously-seen packet payload sizes. In order to boost the resulted performance, we build a Deterministic Finite Automaton (DFA) by unrolling the failure links in advance, adding them as additional transitions directly to the appropriate node. This optimization may expand the size of the automaton; however, it offers substantial performance speedup when a large number of signatures is compiled to a single automaton. In Section 4.2, we present the automaton creation time as well as the automaton size according to the number of signatures and their size.

### 3.2. Implementing with OpenCL

To uniformly execute the pattern matching engine across every device in our testbed machine (i.e., the main processor Intel i7-8700K, a high-end discrete NVIDIA GTX 980 GPU (NVIDIA, Santa Clara, CA, USA) and an Intel UHD Graphics 630 integrated GPU (Intel, Santa Clara, CA, USA)), we utilize the OpenCL framework. Our testbed system runs Arch Linux 4.19.34-1-lts, and we use the Intel OpenCL 2.1 SDK for the Intel devices (i.e., the UHD Graphics 630 GPU and the Intel i7-8700K CPU) and the OpenCL SDK from the NVIDIA CUDA Toolkit 10.2.120 for the NVIDIA GTX 980 GPU. In OpenCL, an instance of a given code block and a thread that executes it is called *work-item*, and a set of multiple work-items is called *work-group*. Different work-groups can run concurrently on different hardware cores. Typically, GPUs contain a significantly faster thread scheduler, thus it is recommended to spawn a large number of work-groups, since it hides the latency that is introduced by heavy memory transfers through the PCIe bus. While a group of threads waits for data consumption, another group can be scheduled for execution. On the other hand, CPUs perform more efficiently when the number of work-groups is close to the number of the available cores. When executing compute kernels on the discrete GPU, the first thing to consider is how to transfer the data to and from the device. Discrete, high-end GPUs have a dedicated memory space, physically independent from the main memory. To execute a task on the GPU, we must explicitly transfer the data between the host (i.e., DRAM) and the device (i.e., GPU DRAM). Data transfers are performed via DMA, so the host memory region should be page-locked to prevent any page swapping during the time that transfers take place. In OpenCL, a data buffer, which is required for the execution of a computing kernel, has to be created and associated with a specific *context*. Different contexts cannot share data directly. Thus, we have to explicitly copy the received network packets to a separate page-locked buffer that has been allocated from the context of the discrete GPU and can be moved towards its memory space via PCIe. Data transfers (host → device → host) and GPU execution are performed asynchronously, permitting a pipeline of computation and communication, something that significantly improves parallelism. Moreover, when the processing is performed on an integrated GPU, expensive data transfers are not required, since both devices have direct access to the host memory. To avoid redundant copies, we explicitly map the corresponding memory buffers between the CPU and the integrated GPU, using the OpenCL’s clEnqueueMapBuffer() function.

**Figure 5:**
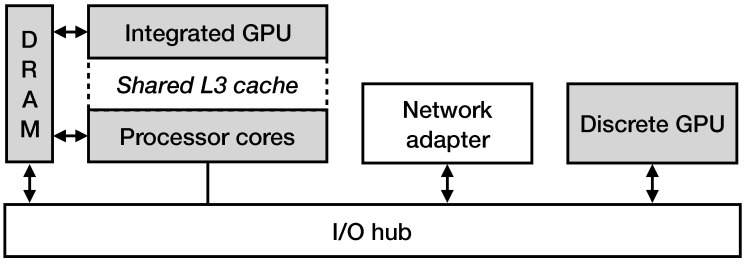
An illustration of the packet processing scheme in a hardware setup that contains one main processor packed in the same die with an integrated GPU and one discrete high-end GPU.

Figure 5 presents an illustration of the packet processing scheme in a hardware setup of a commodity machine that contains one main processor packed in the same die with an integrated GPU and one discrete high-end GPU. As previously explained, to process a network packet on a discrete GPU, the steps are the following: (i) the DMA transaction between the NIC and the main memory, (ii) the transfer of the packets to the I/O bus that corresponds to the discrete GPU, (iii) the DMA transaction to the memory space of the discrete GPU, (iv) the execution of the OpenCL processing kernel and (v) the transfer of the results back to the host memory. Due to the PCIe interconnect inability to quickly handle small data transfers, all data transfers are instructed to operate on large batches. The packet processing on an integrated GPU follows a shorter path, since the integrated GPU and CPU share the same physical memory space, which allows in-place data processing, resulting in lower execution latency.

Memory accesses can be critical to the overall performance sustained by our application. GPUs execute code in a Single-Instruction-Multiple-Threads (SIMD) fashion, meaning that at each cycle multiple threads execute the same instruction. Moreover, they offer support for Single-Instruction-Multiple-Data (SIMD) execution when using vector data types (such as the ushort16 that is able to store 16 16-bit long values), since the vectorized code is translated to SIMD instructions [17]. Furthermore, OpenCL offers the so-called *local memory*, which is a memory region that is shared between every work-item inside a work-group. This local memory is implemented as an on-chip memory on GPUs, which is much faster than the off-chip global memory. Hence, when we execute our engine on GPUs, we can utilize this local memory in order to improve the overall performance.

### 3.3. Packet Processing Parallelization

**Figure 6:**
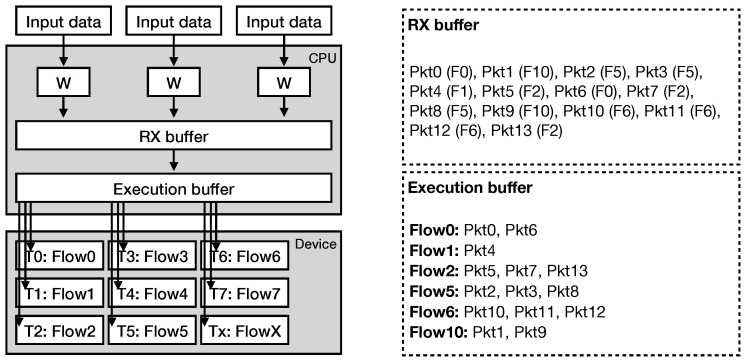
Overview of the packet processing architecture. Each worker thread is assigned to a single input source, transferring the packets into the RX buffer. The RX buffer continuously receives packets, copying its contents to the execution buffer, where the packets are divided into flows, ordered and copied to the device’s memory address space for processing. Each compute kernel thread is assigned to a single flow.

The overall architecture of our intrusion detection system is presented in Figure 6. The system utilizes one or several CPU worker threads, assigning each one to a single input source (e.g., NIC).

Once a CPU thread receives a network packet, it forwards it to a receive buffer, called RX batch (Figure 6). At this point, the receive buffer is filled with packets belonging to one or several TCP flows. When the buffer is full, our system generates an execution batch with the traffic contained in the receive buffer. The execution batch contains the payload sizes of the received network packets, divided and ordered by the corresponding flows. In this way, we transform the input traffic to series of payloads with each series containing information of a single flow, ready to be processed by our pattern matching engine. Then, we transfer the execution batch to the device’s memory address space. In the meantime, the receive buffer continues to accepts incoming packets, avoiding packet losses.

We implement the pattern matching engine of our system as an OpenCL compute kernel. Unlike other relevant works that follow a packet-per-thread processing approach [9,10,18,19], we follow a flow-per-thread approach. This means that each thread reads at least one network flow from the execution batch and then performs the processing (Figure 6). Whenever a batch of packets is received and forward for TCP flow ordering and processing by the device, new packets are copied to another batch in a pipeline fashion. Moreover, in order to fully utilize the SIMD capabilities of the hardware, we represent the payload sizes in the execution buffer as unsigned short integers. In this way, we are able to access the data using the ushort16 vector data type, as described above, in a row-major order, being able to fetch information for 16 packets at once. During the processing, the pattern matching kernel uses one ushort value as input, representing one payload size, at each step in order to traverse the automaton, as described in Section 3.1.

If a signature is identified, the engine reports the suspicious TCP flow identifier, packed with the packets that matched the signature—using the first and the last packet contained in the signature, together with the signature identifier. We encode this information using four ushort values for each TCP flow that is identified as suspicious. In this way, we minimize the amount of data that need to be transferred back from the device to the host’s DRAM. Moreover, in cases where an execution batch does not contain any suspicious flows, the engine does not need to perform any other memory transfers except for initially transferring the data for processing. Finally, in order to provide support for even further analysis, we keep a copy of the packet payload and metadata to the host’s memory until their processing in the GPU has finished so their payloads can be examined in combination with the information provided by the engine.

## 4. Evaluation

In this section, we evaluate this work in two parts. First, we evaluate the effectiveness of the generated signatures, while, in the following paragraphs, we present the performance results of the implemented intrusion detection engine. We chose to use a fraction of the UNSW-NB15 dataset [13] (i.e., “Exploits”, “Reconnaissance” and “DoS” attacks) to evaluate our methodology and the signatures that it produced. We select these three categories (nine categories in total) since they concentrate an important fraction of the network flows that are included in the dataset (more than 60% of the total flows).

### 4.1. Signature Quality

In this section, we demonstrate the expressiveness and effectiveness of the proposed signature specification by generating pattern signatures for a set of malicious activities, evaluating their accuracy. We use 40% randomly chosen flows from the ground-truth dataset as a reference, and the remaining 60% for the evaluation. While the typical split is 70% for training and 30% for testing, we stress the effectiveness of our methodology using only a 40% of the data for training.

In order for the TCP protocol to deliver data reliably, it provides a set of mechanisms to detect and avoid unpredictable network behavior, like packet loss, duplication, or reordering. In our methodology, we choose to discard packets that do not offer substantial information to the flow, like re-transmitted packets, focusing entirely on processing packet metadata. Table 4, Table 5 and Table 6 show the resulting true positive rates (TPR) for different attack types found into the ground truth dataset. Each traffic trace in the test dataset contains a combination of malicious and benign traffic (labeled). When a signature reports malicious activity, we compare it with the actual category of the flow (malicious or benign). If the activity is correctly reported as malicious, then the TP counter is increased. Otherwise, we have a false positive (FP). The true positive rates, as presented in Table 4, Table 5 and Table 6, show the effectiveness of each signature according to the signature’s length. More specifically, the reported true positive rates indicate that the signature length can significantly affect the signature effectiveness. For instance, short signatures result in higher TPR. In some cases, however, a short signature (that results in a high TPR) is possible to introduce the trade-off of resulting in a high FDR as well. The false discovery rates (False discovery rate can be calculated as FDR = FP/(TP+FP)), as presented in Table 7, Table 8 and Table 9, present the percentage of signatures that falsely reported malicious activity. To measure the FDR, we search for the same signatures (as in the previous experiment) against benign traffic.

As it occurs from our results, the FDR rates for the “Exploits” category are low, especially for short signatures, resulting in false discovery rates below 1%. In the “Reconnaissance” category, short signatures result in unacceptable false discovery rates. However, signatures of 8, 10, or 12 packets per sequence, in the same category, result in balanced rates between true positives and false discovery. As it occurs from Table 6 and Table 9, signatures generated to identify DoS attacks perform poorly, both for TPR and FDR. This could potentially prove our claim that building expressive and effective signatures is highly challenging. As part of our future work, we plan to include more packet metadata in the signature generation phase, except for packet payload sizes and direction. Building signatures that present a balanced behavior, both in terms of true and false positives, is probably the optimal solution; however, it is not a trivial procedure.

In the same tables, we present the results while using different kinds of packet metadata; instead of only using the packet payload length as part of the signature, we also use the packet direction (i.e., source to destination, destination to source, and bi-directional). As it occurs, signatures that also indicate the direction result in high true positive rates.

Apparently, signatures’ effectiveness can be further improved to produce less false positives, while keeping the true positive rates high. Taking into account more packet metadata, except for packet payload length and direction, is one way to accomplish this. For instance, more packet and flow metadata could strengthen the results for cases like the DoS attack detection (Table 6 and Table 9). However, in order to implement a simple, flexible and efficient intrusion detection engine (in terms of performance), we choose to generate and test signatures using only the packet payload length and direction.

At this point, we want to stress the fact that the generated signatures are strongly affected by the ground-truth dataset used, which contains the network traffic traces with the malicious and benign activity. Being highly dependent on a rich ground-truth dataset for signature generation, we plan to experiment with more and diverse datasets (for signature generation) and test them against real network traffic environments, in the future.

### 4.2. Performance Micro-Benchmarks

For the performance evaluation of our implementation, we use a commodity high-end machine. The hardware setup of our machine includes an Intel i7-8700K processor with six cores that operate at 3.7 GHz with hyper-threading enabled, providing us with 12 logical cores, configured with 32 GB RAM. The main processor is packed with an Intel UHD Graphics 630 integrated GPU. In addition, we use a NVIDIA GeForce GTX 980 GPU. Our testbed system runs Arch Linux 4.19.34-1-lts, and we use the Intel OpenCL 2.1 SDK for the Intel devices (i.e., the UHD Graphics 630 GPU and the Intel i7-8700K CPU) and the OpenCL SDK from the NVIDIA CUDA Toolkit 10.2.120 for the NVIDIA GTX 980 GPU. At this stage, we only perform offline traffic processing, meaning that the application reads the traffic from memory; thus, for the performance evaluation, we focus on micro-benchmarks in order to present the throughput and latency of the engine’s execution. To measure the performance capabilities of the engine, we used two sets of traffic traces: (i) one set with fully benign etwork flows and (ii) one set with 90% benign network flows and 10% malicious network flows. The malicious network flows contain packet sequences that lead the signatures to report an intrusion event. Since the engine of the intrusion detection system processes batches of network flows (not one flow at a time, but numerous flows in parallel), we performed numerous performance measurements using different number of flows per batch batch. The packet sizes that were contained in each flow were varying, but fixed per flow.

**Figure 7:**
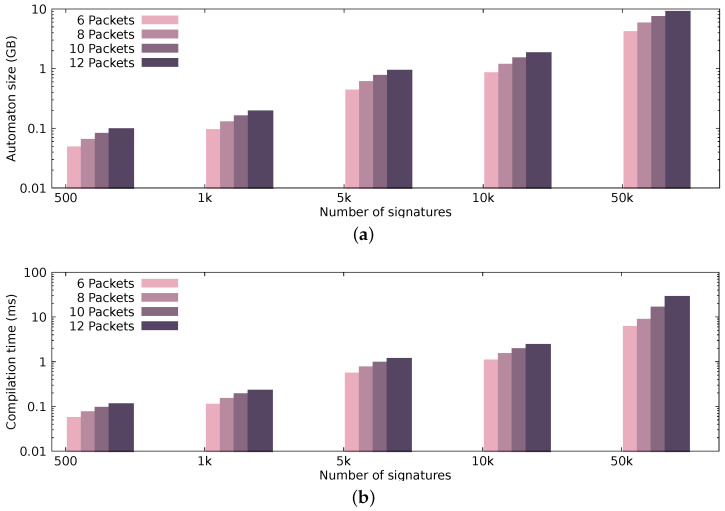
Automaton size and compilation time using different combinations of patterns and pattern sizes. (**a**) Automaton size; (**b**) Automaton compilation time.

**Figure 8:**
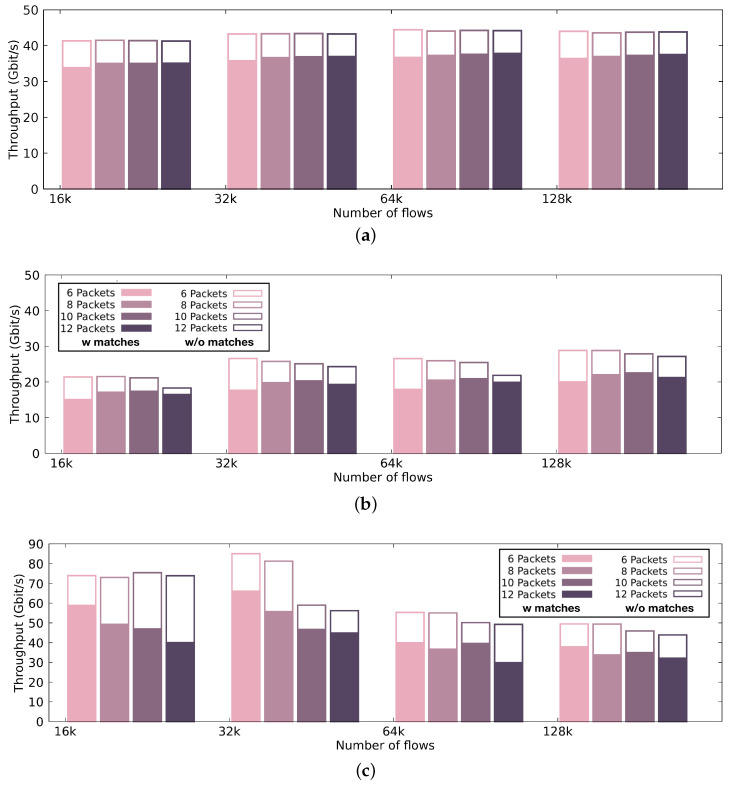
Throughput of our pattern matching engine using a discrete GPU, an integrated GPU and a CPU. Color-filled bars indicate the performance achieved by the pattern matching engine when the selection of (i) signatures and (ii) input results in a computationally loaded condition (i.e., 100% malicious traffic), while the white-filled bars with borders indicate the performance achieved in a computationally relaxed condition (i.e., less than 10% malicious traffic). (**a**) Throughput of the intrusion detection engine using the discrete GPU.; (**b**) Throughput of the intrusion detection engine using the integrated GPU.; (**c**) Throughput of the intrusion detection engine using the CPU.

**Figure 9:**
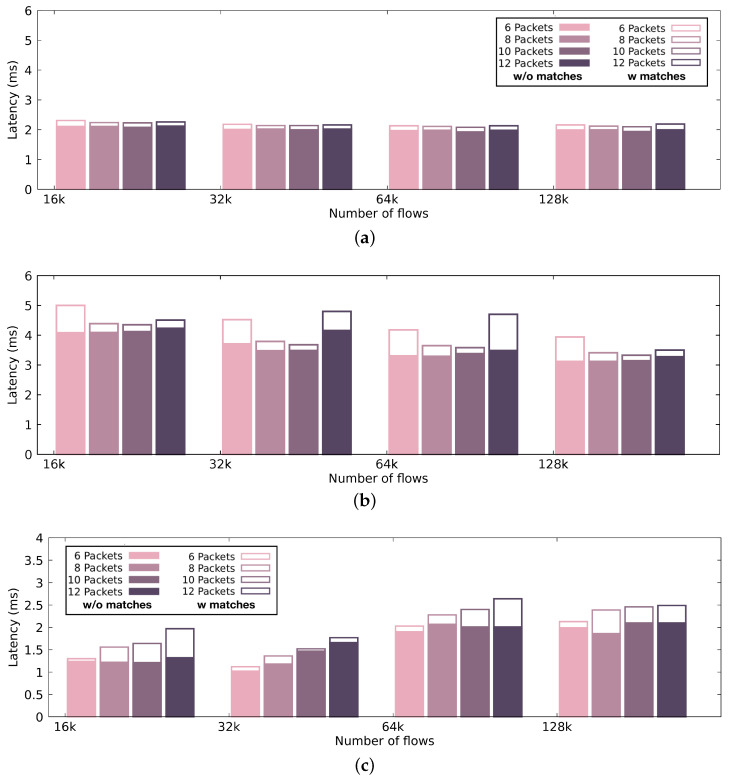
Latency of our pattern matching engine using a discrete GPU, an integrated GPU and a CPU. Color-filled bars indicate the performance achieved by the pattern matching engine when the selection of (i) signatures and (ii) input results in a computationally relaxed condition (i.e., less than 10% malicious traffic), while the white-filled bars with borders indicate the performance achieved in a computationally loaded condition (i.e., 100% malicious traffic). (**a**) Latency of the intrusion detection engine using the discrete GPU.; (**b**) Latency of the intrusion detection engine using the integrated GPU.; (**c**) Latency of the intrusion detection engine using the CPU.

Figure 7 presents the automaton size and compilation time, while Figure 8 and Figure 9 illustrate the performance achieved by the pattern matching engine in terms of processing throughput and latency, respectively. The performance results that are presented in Figure 8 and Figure 9 display the median values occurring after 30 runs per configuration.

#### 4.2.1. Automaton Properties

To present our automaton’s characteristics, i.e., the automaton size and the compilation time, we generate signature sets out of varying packet sequences, each time increasing the number of signatures and the packet sequence length. Figure 7a presents the size of the automaton in regard to different signature sets. More specifically, we present the size of our automaton, using 500, 1K, 5K, 10K, and 50K randomly generated patterns of sequence length 6, 8, 10, and 12 packets; for example, in Figure 7a, the automaton generated from 10,000 signatures, where each signature resembles a sequence of 10 packets, is around 1.5 GB. Figure 7b presents the compilation time of the automaton, again, depending on the same signature sets. As expected, increasing the packet sequence length (even with only a difference of two packets) can significantly affect the automaton size and compilation time. Discrete GPUs have dedicated memory with limited capacity (currently up to 12 GB), since they do not share the same physical address space with the CPU. In hardware setups that utilize discrete GPUs for processing, having small automata is crucial; in comparison to setups, where memory resources are sufficient (i.e., an integrated GPU that shares the same physical address space with the CPU). The compilation time of the automaton does not affect the end-to-end performance negatively, since the compilation happens offline and only once.

From the dataset that we used, we extracted a maximum of 1500 signatures when the sequence size was 12 packets—however, we present how our automaton construction technique performs (in terms of memory and processing time) for a larger number of signatures.

#### 4.2.2. Throughput

Figure 8 presents the performance achieved by our pattern matching engine using different many-core hardware architectures. More specifically, we show the throughput sustained by (i) the discrete GTX 980 GPU (Figure 8a), (ii) the Intel UHD Graphics 630 integrated GPU (Figure 8b), and (iii) the Intel i7-8700K CPU (Figure 8c), executing the pattern matching engine of our intrusion detection solution with 1000 signatures of varying lengths. In Figure 8, the color-filled bars indicate the performance achieved by the pattern matching engine when the selection of (i) signatures and (ii) input results to a computationally loaded condition. In the figure, we present the worst-case scenario, where we have full contamination of the traffic. White-filled bars with borders indicate the performance achieved in a computationally relaxed condition (i.e., less than 10% infected traffic), which is the most realistic scenario. We present the throughput using different packet batch sizes. In general, when we use discrete GPUs, increasing the batch size results in better sustainable throughput. Since, typically, the discrete GPU and the CPU communicate over the PCIe bus, and they do not share the same physical address space, it is preferable that all data transfers operate on fairly large chunks of data, due to the PCIe interconnect inability to handle small data transfers efficiently. The discrete GPU performs with up to 43 Gbps throughput for 10% traffic infection and 37 Gbps throughput for 100% malicious traces. The integrated GPU, which is a less powerful device than the GTX 980 GPU, performs with up to almost 30 Gbps throughput. Executing on the integrated GPU results in a sufficient performance throughput, since expensive memory copies can be prevented. Thus, we also present the throughput across different batch sizes. Unlike GPUs, the main processor performs better for smaller batch sizes, resulting in a maximum throughput of 85 Gbps.

#### 4.2.3. Latency

Through the performance evaluation, it is apparent that, even though discrete high-end GPUs offer high performance rates, the end-to-end performance suffers from the expensive memory copies between the host and the device. In the case of the integrated GPU, we can take advantage of the satisfying computational performance with very low latency in small packet batches, as shown in Figure 9. In Figure 9, the color-filled bars indicate the performance achieved by the pattern matching engine when the selection of (i) signatures and (ii) input, results in a computationally relaxed condition. In the figure, we present the most realistic scenario, where we have less than 10% malicious traffic. White-filled bars with borders indicate the performance achieved in a computationally loaded condition (i.e., 100% malicious traffic), which is the most worst-case scenario. We present the latency using different packet batch sizes. The discrete GPU introduces an almost stable latency across different batch sizes, close to 2 ms. Executing on the integrated GPU results in higher latency records, up to 5 ms. Executing on the main processor adds very low latency—especially for small batch sizes— making it ideal for real-time, latency-intolerant environments. Unfortunately, since there is no equivalent GPU-accelerated implementation for a signature-based intrusion detection engine that searches for packet metadata sequences, we were not able to compare our engine’s performance to another, as a baseline.

## 5. Related Work

In this section, we discuss the state-of-the-art in the domain of network traffic inspection and intrusion detection. Table 10 presents a comparison between this and other related works. In the table, ticks signify that a specific work addresses the corresponding feature/approach, dashes signify that a specific work addresses the corresponding feature/approach, and, finally, circles signify that it is not clear whether the feature/approach is addressed—or to what extent.

Network intrusion detection systems have become very powerful tools in the hands of network administrators and security experts over the past decades, assisting in the detection and prevention of a wide range of attacks. Popular NIDS solutions like Snort [20] and Suricata [21] utilize pattern matching and regular expressions in order to analyze network traffic while Zeek/Bro [22] utilizes scripts that allow easier automation. The research community has also put effort into improving the performance of NIDS using either commodity hardware, such as GPUs [9,23,26,27,28] and parallel nodes [29,30], or specialized hardware, such as TCAMs, ASICs, and FPGAs [31,32,33,34,35]. However, the majority of these works are able to process network traffic that is unencrypted, since they extract meaningful information from network packet payload content. More recently, many works focus on machine learning and deep learning techniques, rather than traditional pattern matching [36,37]. In their work, Sommer and Paxson [38] proposed the use of machine learning for intrusion detection and provide useful guidelines. Shone et al. [39] propose a system that combines deep learning techniques to provide intrusion detection. Tang et al. [40] present a deep learning approach for flow-based anomaly detection in SDN environments, while Niyaz et al. [41] utilize deep learning in order to detect DDoS attacks in such environments. Anderson et al. [42] compare the properties of six different machine learning algorithms for encrypted malware traffic classification. Moreover, Amoli et al. present a real-time unsupervised NIDS, able to detect new and complex attacks within encrypted and plaintext communications [43]. Kitsune [25] is a NIDS, based on neural networks, and designed for the detection of abnormal patterns in network traffic. It monitors the statistical patterns of recent network traffic and detects anomalous patterns. These techniques focus on identifying malicious behavior in the network, examining the characteristics of the underlying traffic, using exclusively machine learning approaches. BlindBox [44] performs deep-packet inspection directly on the encrypted traffic, utilizing a new protocol and new encryption schemes. Taleb et al. [45,46] propose solutions based on per-packet inspection of the header information in order to identify misuses in encrypted protocols. On the other hand, iDeFEND [47] is a framework for inspecting encrypted network data without breaking the security model of end-to-end encryption. Goh et al. [48,49] propose mirroring the traffic to a central IDS, able to decrypt the traffic and perform deep packet inspection, while ProtoMon [50] is based on detection of protocol misuse. Hellmons et al. proposed SSHCure [51], a flow-based intrusion detection system for SSH attacks, while Foroushani et al. [52] propose an approach for detecting anomaly behavior in encrypted access with SSH2 protocol. Many other research and commercial solutions focus on inspection of encrypted network traffic mostly for network analytics [5,24,53]. Lotfollahi et al. [53] present a system that is able to handle both traffic characterization and application identification by analyzing encrypted traffic with deep learning. Conti et al. [5,54] and Taylor et al. [24,55] propose systems that aim to identify user actions and smartphone apps by analyzing Android encrypted network traffic. OTTer [6] is a scalable engine that identifies fine-grained user actions in OTT mobile applications even in encrypted network traffic. Moreover, Rosner et al. [7] present a black-box approach for detecting and quantifying side-channel information leaks in TLS-encrypted network traffic. Symantec offers the Encrypted Traffic Management (ETM) tool [56] that provides visibility into encrypted traffic by decrypting part of the traffic; however, this is a technique that could cause privacy violations. Cisco’s Encrypted Traffic Analytics (ETA) is a proprietary solution [57] for businesses that offers traffic security and analytics by utilizing various features of the network traffic, extracted from other Cisco technologies.

To compare this work with the state-of-the-art, it is very common to encounter works that examine the feasibility of identifying the network traffic class, while encrypted (e.g., a webpage, a mobile application or some malicious activity). As already stressed, all these works focus on machine learning techniques that signify the network-related characteristics that indicate this specific class. However, the majority does not focus on providing a system implementation able to monitor and inspect network traffic in real time. In this work, we aim to advance the state-of-the-art offering an intrusion detection implementation that combines the following: (i) we generate signatures from packet metadata, found exclusively in network packet headers—the information that is available for processing in encrypted network traffic, (ii) we implement a signature-based intrusion detection engine using an extended version of the Aho–Corasick algorithm to support integers—these integers describe packet size sequences, (iii) we enhance our system’s performance using accelerators.

### Traffic Analysis Resistance

As already discussed, properties of network traffic that remain observable after encryption, namely packet sizes and timing, can reveal surprising information about the traffic’s contents. While there are some legitimate uses for encrypted traffic analysis, these techniques raise important questions about the privacy of encrypted communications. A typical approach to mitigate such threats is to pad packets to uniform sizes or to send packets at fixed timing intervals. Wright et al. propose a method for thwarting statistical traffic analysis algorithms by morphing one class of traffic to look like another [58]. Through the use of convex optimization techniques, authors show how to modify packets in real-time to reduce the accuracy of a variety of traffic classifiers while incurring much less overhead than padding. The altered data are then sent to the network stack encrypted and then sent across the network [58]. AnonRep [59] offers anonymity and privacy guarantees for reputation and voting systems. TARN [60] randomizes IP addresses, while TARANET [61] employs packet mixing and splitting for constant-rate transmission. Luo et al. [62] designed the HTTPOS fingerprinting defense at the application layer. HTTPOS acts as a proxy accepting HTTP requests and obfuscating them before allowing them to be sent. It modifies network features on the TCP and HTTP layer such as packet size, packet time, and payload size, along with using HTTP pipelining to obfuscate the number of outgoing packets. They showed that HTTPOS was successful in defending against a number of classifiers. Dyer et al. [63] combine fixed packet sizes and constant rate traffic. Frolov et al. [64] propose uTLS that mimics other popular TLS implementations to prevent censorship. Walkie-Talkie molds burst sequences so that sensitive and non-sensitive pages look the same [65]. There have been efforts to create messaging protocols that provide anonymity and privacy guarantees in the face of traffic analysis [66,67,68,69].

Dissent [66] and Riposte [67] are systems that provide strong guarantees by using message broadcasting. They protect packet metadata but may be unattractive due to scalability issues. Herd [70] is another system that tackles the case of anonymity for VoIP calls, by addressing, like the former proposals, some of the limitations of the more general-purpose Tor anonymity network [71]. Vuvuzela [68] and Atom [69] employ differential privacy to inject noise into observable metadata.

In this work, we present the implementation of a network intrusion detection system that is effective even in encrypted networks. When it comes to intrusion detection, being able to accurately report a suspicious event is crucial. Hence, we believe that typical string pattern matching for packet payload content inspection should be integrated with our proposed packet metadata matching for more mature and effective intrusion detection systems, even if techniques like encryption and traffic analysis resistance can circumvent them.

## 6. Conclusions

Traditional traffic monitoring processes, such as intrusion detection and prevention tools, use deep packet inspection techniques that often focus on examining the packet payload contents to report certain activities, resulting in poor adaptiveness for encrypted network traffic. In this work, we propose a fast signature-based intrusion detection engine tailored for encrypted network traffic. Specifically, this work is divided into two parts: (i) the signature generation and (ii) the implementation of the intrusion detection engine. The signatures are generated using only network packet metadata, strictly extracted from packet headers, an approach tailored for encrypted networks. For the engine implementation part, we use OpenCL to allow uniform execution across GPU accelerators and a high-end CPU. Using GPUs, the intrusion detection engine can perform with up to 37 Gbps throughput in the worst-case scenario (100% malicious traffic) and with up to 43 Gbps throughput in a computationally relaxed condition (less than 10% malicious traffic). In addition, we achieved a performance of up to 85 Gbit/s throughput using a commodity high-end CPU. As part of our future work, we plan to include other network packet metadata in the signature generation phase (besides packet payload sizes and direction) and investigate how they could reveal the encrypted network nature even in traffic analysis resistant implementations.

## Figures and Tables

**Table 1:** Simple signature formats.

Direction	Signature Specification
Src → Dst	64,100,109,109,109,5
Src ↔ Dst	64,−0,100,−100,109,−30,109,…

**Table 2:** Regular-expression-like signature formats.

Direction	Signature Specification
Src → Dst	64–69,100,109{2–3},5
Src ↔ Dst	64–69,−0,100,−100,109,−30,109,…

**Table 3:** A subset of the attributes and their descriptions that characterize a single attack record in the dataset [15].

Name	Description
srcip	Source IP address
sport	Source port number
dstip	Destination IP address
dsport	Destination port number
proto	Transaction protocol
state	Protocol state
dur	Record total duration
sbytes	Source to destination bytes sent
dbytes	Destination to source bytes sent
service	e.g., http, ftp, smtp, ssh, dns
sload	Source bits per second
dload	Destination bits per second
spkts	Source to destination packet count
dpkts	Destination to source packet count
attack_cat	Name of attack
label	0 for benign, 1 for attack records

**Table 4:** Resulting true positive rate (TPR) of different types of signatures against the tested traffic. Signatures and traffic are part of the attack samples from the “Exploits” category. The signatures are generated after a combination of packet direction and packet payload size.

	Packet Sequence Length				
**Direction**	**4**	**6**	**8**	**10**	**12**
Source → Destination	100%	93%	69%	63%	54%
Destination → Source	100%	55%	37%	30%	30%
Source ↔ Destination	100%	100%	97%	74%	61%

**Table 5:** Resulting true positive rate (TPR) of different types of signatures against the tested traffic. Signatures and tested traffic are part of the attack samples from the “Reconnaissance” category. The signatures are generated after a combination of packet direction and packet payload size.

	Packet Sequence Length				
**Direction**	**4**	**6**	**8**	**10**	**12**
Source → Destination	100%	89%	89%	89%	87%
Destination → Source	100%	98%	74%	70%	68%
Source ↔ Destination	100%	100%	89%	86%	82%

**Table 6:** Resulting true positive rate (TPR) of different types of signatures against the tested traffic. Signatures and tested traffic are part of the attack samples from the “DoS” category. The signatures are generated after a combination of packet direction and packet payload size.

	Packet Sequence Length				
**Direction**	**4**	**6**	**8**	**10**	**12**
Source → Destination	100%	61%	49%	44%	10%
Destination → Source	100%	50%	26%	14%	13%
Source ↔ Destination	100%	100%	72%	27%	15%

**Table 7:** Resulting false discovery rate (FDR) of different types of signatures against part of the benign traffic. Signatures are part of the attack samples from the “Exploits” category. The signatures are generated after a combination of packet direction and packet payload size.

	Packet Sequence Length				
**Direction**	**4**	**6**	**8**	**10**	**12**
Source → Destination	0.8%	0.7%	0.6%	0.2%	0.1%
Destination → Source	0.7%	0.5%	0.05%	0%	0%
Source ↔ Destination	0.8%	0.8%	0.6%	0.2%	0%

**Table 8:** Resulting false discovery rate (FDR) of different types of signatures against part of the benign traffic. Signatures are part of the attack samples from the “Reconnaissance” category. The signatures are generated after a combination of packet direction and packet payload size.

	Packet Sequence Length				
**Direction**	**4**	**6**	**8**	**10**	**12**
Source → Destination	62%	0.9%	0.7%	0.3%	0%
Destination → Source	65%	15%	0.1%	0.5%	0.3%
Source ↔ Destination	63%	63%	0.11%	0.1%	0%

**Table 9:** Resulting false discovery rate (FDR) of different types of signatures against part of the benign traffic. Signatures are part of the attack samples from the “DoS” category. The signatures are generated after a combination of packet direction and packet payload size.

	Packet Sequence Length				
**Direction**	**4**	**6**	**8**	**10**	**12**
Source → Destination	62%	43%	30%	21%	18%
Destination → Source	65%	15%	10%	0.5%	0.3%
Source ↔ Destination	63%	63%	27%	0%	0%

**Table 10:** Comparison of recent network traffic inspection and intrusion detection works.

	Offline Analysis	Encrypted Traffic	Online Inspection	Performance Efficiency	Details
Snort [20], Suricata [21], Zeek/Bro [22]	✓	∘	✓	∘	Signature/Anomaly-based, Payload inspection, Network security
Gnort [23], MIDeA [9]	∘	—	✓	✓	Signature-based, Payload inspection Network security, GPU-acceleration
Conti et. al [5], Taylor et. al [24], Profit [7]	✓	✓	—	—	ML-based, Network analytics
OTTer [6]	✓	✓	✓	✓	Signature-based, Pattern mining, Network analytics
Profit [7]	✓	✓	—	—	ML-based, Network analytics
Kitsune [25]	✓	✓	✓	—	Neural Network, Network security
**HeaderHunter**	✓	✓	✓	✓	Signature-based, Network security, GPU-acceleration

## Data Availability

Publicly available datasets were analyzed in this study. This data can be found here: https://www.unsw.adfa.edu.au/unsw-canberra-cyber/cybersecurity/ADFA-NB15-Datasets/ (accessed on 4 February 2021).
